# Feasibility of the inhibitor development for cancer: A systematic approach for drug design

**DOI:** 10.1371/journal.pone.0306632

**Published:** 2024-08-22

**Authors:** Yu Jiang, Ling Liu, Yichao Geng, Qingsong Li, Daxian Luo, Li Liang, Wei Liu, Weiwei Ouyang, Jianping Hu

**Affiliations:** 1 Key Laboratory of Medicinal and Edible Plants Resources Development of Sichuan Education Department, School of Pharmacy, Chengdu University, Chengdu, China; 2 Department of Thoracic Oncology, Affiliated Cancer Hospital, Guizhou Medical University, Guiyang, China; Universidade Federal do Para, BRAZIL

## Abstract

The traditional Chinese medicine (TCM) bupleurum-ginger-licorice formula presents significant anti-cancer effects, but its active ingredients and inhibitory mechanism remain unclear. In this work, the core effective ingredient quercetin and its signal transducer and activator of transcription 3 (Stat3) receptor both were identified by network pharmacology. Quercetin is a low-toxicity, non-carcinogenic flavonoid with antioxidant, anti-inflammatory and anticancer activities, which is widely distributed in edible plants. Stat3 can bind to specific DNA response elements and serves as a transcription factor to promote the translation of some invasion/migration-related target genes, considered as a potential anticancer target. Here, molecular docking and molecular dynamics (MD) simulation both were used to explore molecular recognition of quercetin with Stat3. The results show that quercetin impairs DNA transcription efficiency by hindering Stat3 dimerization, partially destroying DNA conformation. Specifically, when the ligand occupies the SH2 cavity of the enzyme, spatial rejection is not conductive to phosphokinase binding. It indirectly prevents the phosphorylation of Y705 and the formation of Stat3 dimer. When the inhibitor binds to the DT1005 position, it obviously shortens the distance between DNA and DBD, enhances their binding capacity, and thereby reduces the degree of freedom required for transcription. This work not only provides the binding modes between Stat3 and quercetin, but also contributes to the optimization and design of such anti-cancer inhibitors.

## Introduction

The nature of cancer is the continuous and uncontrolled proliferation of cells, usually caused by increased conversion of proto-oncogenes to oncogenes or by attenuated mutations in tumor suppressor genes [1]. According to the World Health Organization’s (WHO) annual report for 2023, there were 25.6 million new cancer cases and 12 million cancer deaths globally, making it the second leading cause of death after cardiovascular disease [2]. The incidence of six of the top 10 cancers continues to increase, of these, breast cancer is by far the most commonly diagnosed malignant tumor, with an estimated 2.3 million new cases (11.7%), followed by lung cancer (11.4%), colorectal cancer (10.0%), prostate cancer (7.3%), stomach cancer (5.6%), and liver cancer (4.1%) [3,4]. Clinical strategies for cancer generally require comprehensive treatment, including radical surgery, radiotherapy, chemotherapy, endocrine therapy, targeted drug therapy, etc. Nevertheless, these treatments have certain side effects that are difficult to avoid [3]. Therefore, the development of new cancer therapies less harmful to human body has attracted more and more attention of pharmaceutical chemists and become one of the goals of clinical treatment.

Due to the relatively low toxicity and side effects, the use of traditional Chinese medicine (TCM) materials shows a certain clinical application value. Xiaochaihu decoction mainly consists of the core natural herbs bupleurum, ginger and licorice as a well-known prescription for cancer treatment [5]. The three natural products are rich in flavonoids, which may be the key factor to obvious anticancer activities. Bupleurum has been widely used since ancient times, whose active components may be involved in regulating immune function, enhancing hematopoietic recovery of bone marrow and also inducing apoptosis of cancer cells. In addition, by reducing the side effects of chemotherapy, psychological and physiological stress reactions during treatment can be minimized, thereby improving treatment efficiency [6]. Ginger is rich in various phenolic compounds, such as gingerols and shogaols. Ginger not only has antioxidant, anti-inflammatory, antibacterial, antiemetic and other biological functions, but also its alcohol extract can significantly inhibit the proliferation of cancer cells [7]. Licorice has been used for more than 2,000 years to treat stomach, liver and respiratory diseases. It is the main ingredient of several proprietary Chinese medicines with anticancer activity certified by the National Medical Products Administration (NMPA) [8]. Quercetin is found in the roots, stems, leaves, flowers, and fruits of many plants as an anticancer flavonoid [9–12]. An appropriate intake of low toxicity dietary flavonoid compounds including quercetin can help reduce the risk of melanoma. By using a mouse model to simulate the treatment of melanoma with quercetin, Cao et al. demonstrated that the activated signal transducer and activator of transcription 3 (Stat3) signaling pathway is a candidate target. In principle, quercetin inhibits the activation of Stat3 signal by interfering with phosphorylation and nuclear localization, making it potential to play a role in the prevention and treatment of melanoma [13]. In addition, Zhu et al. investigated the protective effect of quercetin on alcoholic liver injury (ALD) in a mouse model. The results show that the small molecule exerts antioxidant, anti-inflammatory and anti-apoptotic effects through Stat3, nuclear factor (NF)-κB and protein kinase B (Akt) pathways, and inhibits total aspartate aminotransferase, several cytokines, and nitric oxide synthase [14]. Based on human-related cell experiments, Jonathon et al. demonstrated that quercetin can decrease the proliferation and migration of glioblastoma cells by reducing the activation of glycoprotein 130 (gp130), Janus Activated kinases (JAK) and Stat3, suggesting a therapeutic potential for Stat3-active tumors [15]. In addition, quercetin inhibits the infection ability of Epstein-Barr virus (EBV) and also attenuates the resistance to drug therapy, by activating Stat3 receptor and reducing the levels of interleukin-6 (IL-6) and reactive oxygen species (ROS), key factors in B-cell survival [16]. In a word, quercetin, as a dietary flavonoid compound, has been adopted in the treatment of various diseases including cancer.

**Fig 1:**
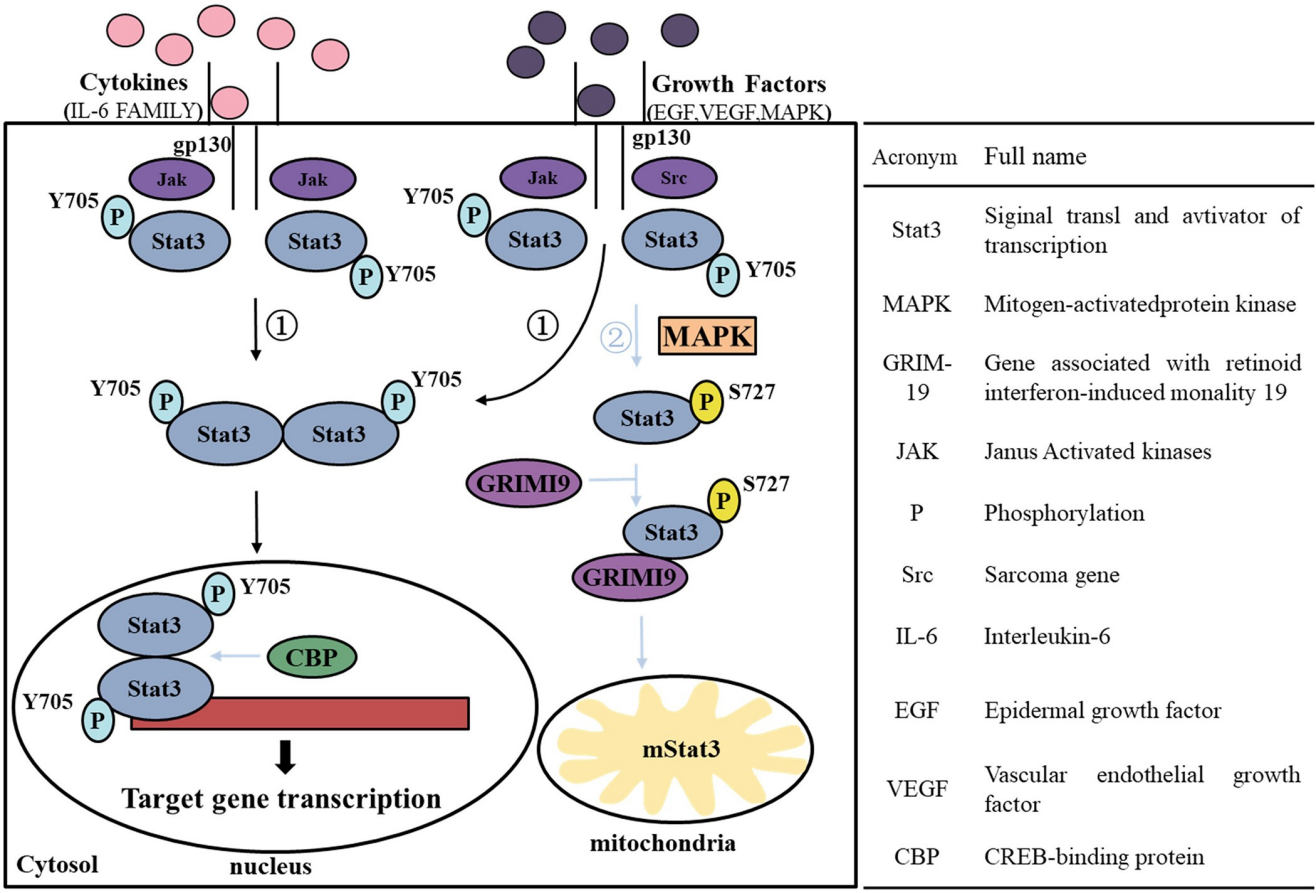
Stat3 participating in a variety of biological functions as a key signaling protein.

The onset and development of tumors is a multifactorial and multistep process, and the breakthrough in their treatment lies in finding effective and specific targets [1,17]. Stat3 is involved in the regulation of cell growth, differentiation, apoptosis and other physiological pathways (see Fig 1) and has become a major target in cancer [18]. When activated by phosphorylation, Stat3 is translocated into the nucleus and plays a role in the transcription of specific target genes [19–21]. Specifically, phosphorylation on Y705 of Stat3 is mainly regulated by Janus-Activated kinases (JAK); subsequently, phosphorylated Stat3 enters the nucleus and forms homo- or heterologous dimers, acting as transcription factors to bind with promoters of target genes and induce transcription [22–26]. In addition, the phosphorylation at S727 of Stat3 is associated with non-transcriptional mitochondrial relocation, which increases cellular respiration and renin-angiotensin system (RAS) transformation by activating the electron transport chain complex [27–31]. Stat3 shares the same sequence and structural homology with other members of the STAT family and is composed of six domains: N-terminal domain (ND, G1-Q136), Frizzled-coiled domain (CCD, V137-A320), DNA-binding domain (DBD, F321-S465), linker subdomain (LD, N466-I585), Src homology 2 domain (SH2, M586-R688), and transcriptional activation domain (TAD, A689-P770) [32,33]. Among these, both the SH2 and DBD domains have been extensively and thoroughly studied. Stat3 dimerization occurs between the phosphopeptide-containing Y705 in one monomer and SH2 in the other monomer. Obviously, inhibition of the SH2 domain will prevent Stat3 dimerization and has become an important strategy for inhibitor design [34]. Phosphorylated Stat3 dimers then enter the nucleus and bind to DNA through the DBD region, ultimately promoting the activation of transcription of a series of genes [35]. DNA ligases are important components of the DNA replication and DNA damage repair pathways. These pathways are not only extremely important for genomic stability of normal cells, but are also extensively involved in cancer development. Thus, selective inhibition of DNA ligases induces apoptosis in malignant cells or inhibits tumor cell proliferation. However, in DNA ligases, the interaction of DBD with the minor groove of the DNA adenylation structural domain (ADD) and the oligonucleotide/oligosaccharide-binding fold (OBold) of DNA ligase I, which would have stimulated the DNA end-joining activity of the catalytic core, and inhibition of 50-phosphate transfer of AMP from ligase to nicked DNA by targeted inhibition of the DBD region. The DBD region of Stat3 consists of eight β-folds, all of which are functional regions that aid in DNA transcription. This shares the same structural domains as human DNA ligase, making it an effective approach by directly targeting DBD. Inhibitors are mainly structure-based drug-designed human DNA ligase inhibitors, natural product inhibitors and novel specific inhibitors [36]. In other words, targeting the DBD domain to prevent Stat3 from interacting with DNA is another way to avoid tumorigenesis.

Some natural compound inhibitors have been previously reported to target nuclear transcription of Stat3. Zhao et al. verified anticancer effect of natural compound alpinetin on ovarian cancer SKOV3 cells using cell experiments. The compound significantly inhibited the formation of SKOV3 cell colonies and spheroids in a dose- and time-dependent manner, ultimately inducing apoptosis. In addition, alpinetin may also exert anticancer effects by inhibiting Stat3 signaling pathway, as reflected in down-regulation by p-Stat3 and decreased expression of the downstream factors c-myc [37]. Using Western blot experiments, Ko et al. found that breast cancer stem cells (BCSCs) are associated with chemotherapy resistance and tumor recurrence. Citrinin inhibits the formation and proliferation of BCSCs, and then induces their apoptosis, which is accompanied by a decrease in the frequency of the cyclin-dependent 44+/ 24- (CD44+/ CD24-) phenotype. In terms of the mechanism of action, Citrinin obviously reduces the total phosphorylation level of the Stat3 pathway, which qualifies as a potential natural anti-breast cancer compound [38]. Xu et al. investigated the potential inhibitory mechanism of cucurbitacin against gastric cancer in vitro and in vivo through cytotoxicity, viability and invasion experiments. According to the pull-down experiment, cucurbitacin reduces the proliferation of gastric cancer cells by decreasing phosphorylation of Y705, and is considered to be an effective lead compound targeting Stat3 [39]. The effects of luteolin on activated human and rat hepatic stellate cells LX-2 and hepatic stellate cells (HSC)-T6 were investigated by Western blot and immunofluorescence experiments. The results showed that luteolin not only significantly decreased two cell viability in a time- and dose-dependent manner, but also attenuated phosphorylation level of Stat3 and expression of regulated proteins c-MYC and cyclin-D1 [40]. The above experiments demonstrate the effectiveness of key components of Chinese herbal medicine regarding in the targeted treatment of cancer.

As mentioned above, natural compounds have been widely used in cancer treatment due to their low toxicity and low carcinogenesis rate, some of which act on the Stat3 target. As a common anti-cancer traditional Chinese medicine (TCM) formula, what are the core substances and action target of the bupleurum-ginger-licorice? What are the mechanisms of recognition and possible inhibition between core active substances and action target? In view of the two important scientific questions above, systematic experiments including network pharmacology, molecular docking, molecular dynamics (MD) simulation, and statistical calculation were conducted in this work. This work not only identified the anticancer core substances and potential targets for the three natural products, but also provided atomic-level molecular recognition details, which helped to explore the inhibitory mechanism and compatibility strategy of TCM.

## Materials and methods

### Network pharmacology

Network pharmacology has been widely applied into the field of TCM, which can effectively predict the core components, action targets, toxicity and side effects of drugs [41]. Specifically, after searching the relevant databases, six main steps were performed: (1) To determine the chemical composition of the TCM, this study first collected the chemical structures and potential targets of the active ingredients in the TCM bupleurum-ginger-licorice formula from the TCMSP database (http://lsp.nwu.edu.cn/tcmsp.php); In this integrated pharmacophore matching database with frequent updates, target compounds were screened by respectively setting oral bioavailability (OB) and drug likeness (DL) scores greater than 30% and 0.18, and the same molecules were removed by structural overlap [42]. With the Retrieve/ID mapping tool (https://www.uniprot.org/), the IDs appearing more than once in Traditional Chinese Medicine Systems Pharmacology Database and Analysis Platform (TCMSP) were converted to UniProtKB identifiers for subsequent enrichment analysis. The target names obtained in TCMSP are converted into the corresponding gene names by comparing human genetic information; (3) Disease targets were collected according to the scope of application of the formula and searched in the "GeneCards" (https://www.genecards.org/) database. The gene targets for the above diseases were processed and duplicates were removed to get the disease targets; (4) Identify common targets for components and diseases using Venn diagrams (https://bioinfogp.cnb.csic.es/tools/venny/), and imported the common targets into the String online platform database (https://string-db.org/) to construct a protein-protein interaction (PPI) network; (5) The core targets obtained from screening were subjected to (Kyoto Encyclopedia of Genes and Genomes, KEGG pathway enrichment analysis. P < 0.01 was set, and the results of KEGG pathway enrichment analysis were visualized using the Microbiology Trust platform (http://www.bioinformatics.com.cn/login/); (6) Network pharmacology results can be visualized through Cytoscape3.9.0—an open-source software for molecular interaction networks and biological pathways [43].

### Molecular similarity

Compounds with similar structures tend to have similar physicochemical properties and biological activities. The principle of molecular similarity is of great value in the field of virtual screening and molecular design of drugs [44,45]. The Open Babel package has been widely adopted by pharmaceutical chemistry experts. It not only enables the conversion, analysis, and storage of modeling, chemistry, and biology data, but also provides a complete and extensible programmer’s toolkit for developing cheminformatics software. In this work, the Open Babel package was used for similarity search of molecular fingerprints, measured by shape function in Eq (1):

$$Shape(f,g)=\sqrt{\int [f{\left(x,y,z)-g(x,y,z)\right]}^{2}dv}$$ (1)

where *f* and *g* are characteristic functions to present the 3D atomic structure of each compound [46–48]. The similarity score ranges from 0 to 1, in which 0 and 1 respectively correspond to no similarity and same molecules. Here, 23 clinically common anticancer agents acting on different targets were selected for similarity analysis with quercetin to predict its potential targets [49].

### Molecular docking

The structure of small molecule quercetin was constructed by ChemBio3D Ultra 14.0 (Cambridge Soft, Cambridge, MA). Energy minimization was performed for quercetin based on MM2 force field with convergence criteria of root mean square (RMS) less than 0.0001 kcal·mol^-1^·A^-1^. The energy-optimized molecule is then individually docked into the receptor’s binding pocket with AutoDock4.2 package adopting the Lamarck Genetic Algorithm (LGA) [50], which estimates near-natural complex conformation based on semi-empirical free energy. The semi-empirical free energy is composed of four terms, namely the intramolecular energy, the van der Waals interaction, the H-bond interaction and the electrostatic interaction. In order to effectively consider structural flexibility of small molecules, all rotations of single bonds are fully sampled during molecular docking. A total of 100 inhibitor conformations were collected for each docking experiment, and the snapshot with the lowest energy in the largest cluster was defined as the near natural complex model.

#### Molecular dynamics simulations

Based on Newtonian mechanics, molecular dynamics (MD) simulation is one of the most important techniques for exploring the thermodynamic and kinetic properties of biomolecules [51–55]. In this work, four independent MD simulations at 300 K were performed for the Stat3, Stat3_Lig, Stat3_DNA and Stat3_DNA_Lig systems with AMBER20 package [53], where OL15 and ff14SB force field parameters were respectively adopted for the nucleic acid and protein atoms [56,57]. As with the AMBER position, the use of the gaff force field applies to ordinary organic small molecule force fields. The four investigated systems need to be explained: the Stat3/ Stat3_DNA systems are obtained by removing/ reserving DNA coordinates based on crystal structure (PDB ID: 6QHD); the docking results of two molecules respectively with quercetin are defined as Stat3_Lig and Stat3_DNA_Lig. On the premise that all crystal water molecules were retained, all solutes were dissolved in octahedral box with the boundary of 15.0 Å using the TIP3P water model [58]. Before MD simulation, there are two steps of energy optimization as follows: (1) the solute was constrained with the force constant of 2.09 × 10^5^ kJ mol^-1^·nm^-2^, containing 5000 steps of steepest descent and 5000 steps of conjugate gradient minimization; (2) After removing the geometry constraints, the second optimization was also composed of 20,000 steps of steepest descent and 20,000 steps of conjugate gradient minimization. The convergence criterion of minimization is energy difference of neighboring conformations less than 4.182 × 10^-4^ kJ mol^-1^·nm^-2^.

The integration step was set as 2 fs, and conformational snapshots were collected every 100 ps, resulting in a total of 10,000 conformations during each 100 ns MD simulation. In these simulations, the molecular structures were simultaneously monitored with the VMD1.9.3 package and the trajectory analysis were performed by CPPTRAJ [59,60].

### Binding free energy calculation

Binding free energy can be effectively used to judge molecular recognition between receptor-ligand, and is also a key criterion for evaluating the activity of drug molecules. Molecular mechanics/ Poisson Boltzmann (MM/PBSA) method was used to predict the binding free energy based on MD trajectories of the Stat3, Stat3_Lig, Stat3_DNA and Stat3_DNA_Lig systems. Total 100 conformations were collected from each systematic MD trajectories every 1 ns intervals from 1 to 100 ns. MM/PBSA has been widely used to estimate protein-ligand binding affinity due to its efficiency and stability highly correlated with experimental results [61]. The formula is as:

$${\Delta G}_{bind}=\Delta H-T\Delta S=({\Delta E}_{VDM}+{\Delta E}_{ELE}+{\Delta G}_{PBELE}+{\Delta G}_{PBSUR})-T\Delta S$$ (2)

where Δ*E*_VDM_ indicates the intramolecular Van der Waals energy under vacuum, while Δ*E*_ELE_ refers to the electrostatic fraction. Δ*G*_PBELE_ and Δ*G*_PBSUR_ represent the hydrophilic and hydrophobic sections of the solvation binding free energy, respectively. Δ*H* corresponds to the total enthalpy change and *T*Δ*S* is the product of conformational entropy and ambient temperature.

The Solvated interaction energy (SIE) method combined with MD simulation of the trajectories, the binding free energies of proteins and inhibitors can be rapidly predicted. The equation for calculating the binding free energy is given below:

$$\Delta G_{bind}(\rho ,D_{in},\alpha ,\gamma ,C)=\alpha [E_{c}(D_{in})+\Delta G_{bind}^{R}(\rho ,D_{in})+E_{vdw}+\gamma \Delta MSA(\rho )]+C$$ (3)

Δ*G*_*bind*_(*ρ*, *D*_*in*_, *α*, *γ*, *C*) is the binding free energy, which is related to the parameters *ρ*, *D*_*in*_, *α*, *γ*, *C*, respectively; The parameter α is the "global scaling factor", which characterizes the loss of conceptual entropy due to binding, and is set to 0.1048; E_c_(D_in_) is the "Coulomb interaction of molecules", where D_in_ is the "internal dielectric constant of the solute", set to 2.25; $\Delta G_{bind}^{R}(\rho ,D_{in})$ is the change in reaction field energy, utilized in the boundary element program BRI BEM, where *ρ* is the linear proportionality constant of the van der Waals radius 1.1; *E*_*vdw*_ is the van der Waals interaction of the molecules; *γ* is the molecular surface area coefficient, set to 0.0129kcal/mol·A^2^; Δ*MSA* is the change in molecular surface area; C is the calibration constant (because SIE calculations are generally overestimated by a little bit), set to -2.89 kcal/mol.

#### Free energy landscape

Free energy landscape (FEL) [62] is mainly used to study molecular motions and conformational changes for biological systems, by comparing the minimum value of free energy surface (corresponding to the most stable state of the system) and demarcation points (related to a transient state in the process of conformational transition). The lowest free energy region represents a range of steady state conformations in the system under study under physiological conditions [63]. The free energy barrier is used to represent the difficulty of transition between different steady states. In FEL analysis, the required conformational sampling is based on principal component analysis (PCA) [61] on MD trajectories. According to Boltzmann distribution, the probability of a reaction coordinate is obtained, and then converted into the free energy potential energy surface. It is defined as follows:

$$\Delta G(X)={-k}_{B}T\;\ln\;P(X)$$ (4)

Where *X* represents a particular principal component (PC), *k*_*B*_ is the Boltzmann constant, and *T* expresses the absolute temperature in Kelvin; *P*(*X*) is the probability of conformational distribution, representing the contribution of a particular conformation to the overall PCs.

#### Cluster analysis

Cluster analyses were performed for 10,000 snapshots obtained from each MD simulation using MMTSB [64] software package. The basic idea is to calculate root mean square deviation (RMSD) values of Cα atoms between various conformations, and establish N × N RMSD matrix, where N is the number of snapshots. Assuming a RMSD threshold, if the RMSDs between two arbitrary conformations are smaller than this threshold, they are grouped into one certain cluster, in which the lowest energy snapshot is taken as the representative conformation. It can correspond exactly to the free energy landscape of the four investigated Stat3 systems.

### Weak interactions and conformational analysis of DNA

The interactions within biological macromolecules are mainly divided into chemical bonds and non-covalent bonds. Ionic bond is one of the important covalent bonds with high bonding strength. Non-covalent bonds possess relative lower energy, also known as the weak interactions, which mainly include H-bond/ halogen bond/ pi-pi stack/ Van der Waals forces/ steric hindrance. For the atoms in molecules (AIM) theory, electron density ρ(r) is one of the important indicators to measure the strength of molecular interaction. As a global quantity, ρ(r) can be visualized with the help of the real space function. MP2/6-311+G** is often used to calculate the reduced density gradient (RDG) isosurface representing the weak interaction region [65]. The formula for calculating RDG is as follows:

$$RDG=\frac{1}{{2\times (3\times \pi^{2})}^{\frac{1}{3}}}\times \frac{\left|\nabla \rho (\gamma )\right|}{{\rho (\gamma )}^{\frac{4}{3}}}$$ (5)

where ∇ is the gradient operator and |∇ρ(r)| is the magnitude of electron density gradient. In this work, based on RDG theory and free-electron density approximation, the changes of weak non-covalent interactions for double-stranded nucleic acid sequences were compared after the association with quercetin via multiwfn software package [66].

### Conformational analysis of DNA

Most of the DNA was in the classical Watson-Crick model, namely B-form DNA. While in different solution environments or after association with different substrates, its conformation changes significantly, showing structural and functional diversity. As a widely used nucleic acid conformational analysis program, Curves provides complete DNA structure parameters, including base pair axis, intra-base pair, inter-base pair, backbone and groove width [67]. In general, the major and minor grooves of DNA are the preferred binding sites for protein-DNA recognition. In this work, comparative Curves analyses were performed for the Stat3_DNA and Stat3_DNA_Lig systems, and 50 snapshots were extracted from each trajectory every 2 ns in order to get the mean DNA conformational parameters. In order to explore DNA structural change after binding quercetin, the parameters including inclination angle, twist angle and groove widths were analyzed emphatically.

## Results

### Determination of anticancer targets

**Fig 2:**
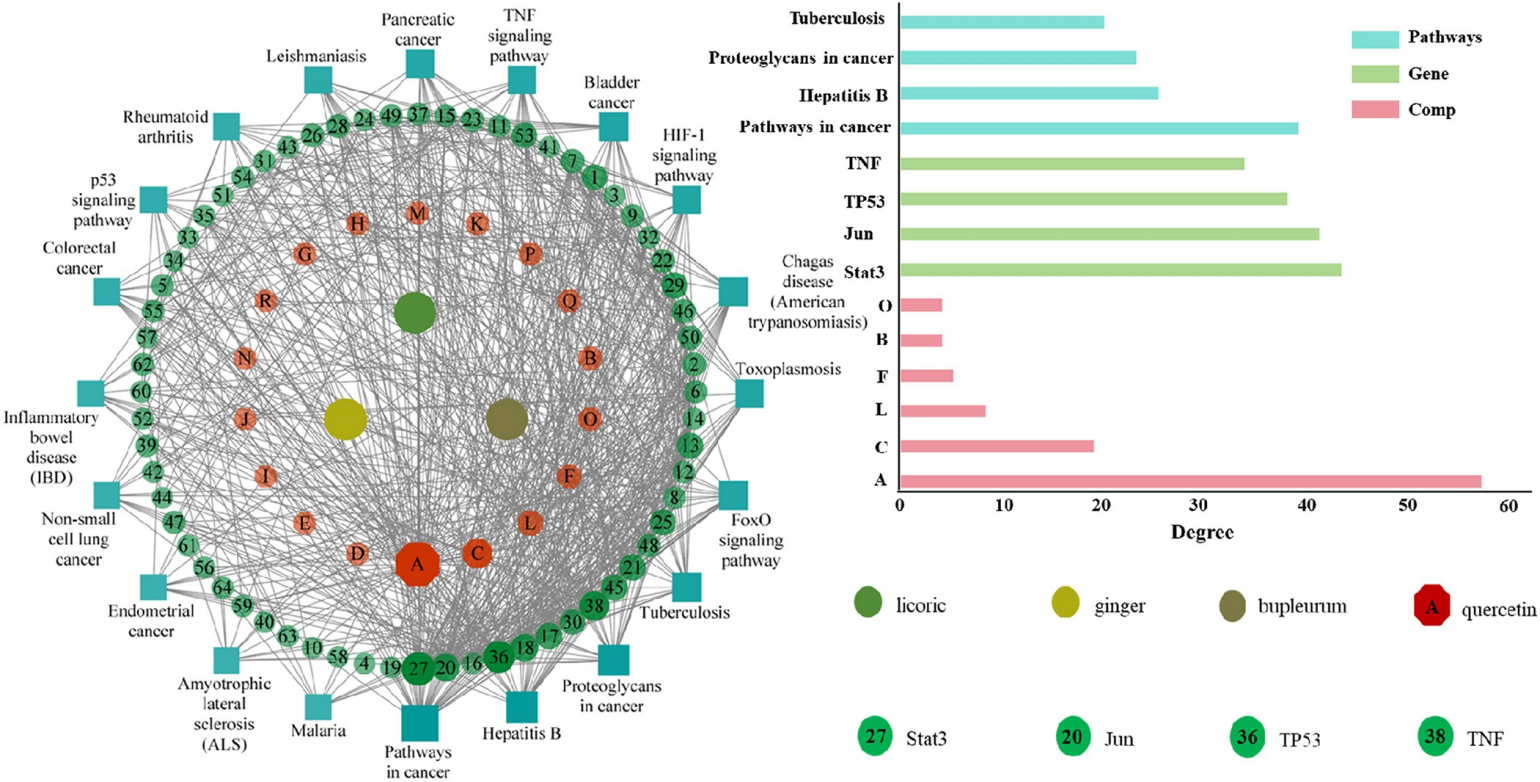
Ingredient-target-pathway network construction of TCM bupleurum-ginger-licorice formula with Cytoscape-3.9.0 software package. The three center points are used to indicate TCM components; the outward red (see S1 Table in S1 File) and green spheres are corresponding to active small molecules and possible targets, respectively; the signal pathways are represented in the outermost blue square; filtering by Degree value greater than median value.

TCM formula has a variety of active components acting on different targets respectively, and finally exert synergistic clinical efficacy. Fig 2 shows the ingredient-target-pathway network connection of TCM bupleurum-ginger-licorice formula, where there are 102 nodes and 618 edges. As seen from Fig 2, the small molecule most closely related to disease targets is quercetin in the formula, which may be the core active ingredient in therapy for cancer. In fact, quercetin indeed has good oral availability and drug-ability. After using OB ≥ 30% and DL ≥ 0.18 as the screening conditions and deleting duplicates, 175 active ingredients were obtained from the three herbs. Chronic hepatitis, cirrhosis, chronic gastritis and pancreatic cancer were used as disease keywords and 904 targets were obtained after removing duplicates. 104 intersecting genes through the online Venny website (S1A Fig in S1 File). The obtained intersecting targets are imported into the String platform to construct a PPI network, and Cytoscape analyzes this PPI network data. Computational screening yielded greater than twice the median degree of targets for this network: Stat3, Jun, TP53, and TNF, and these core targets may be bupleurum-ginger-licorice for the treatment of the above diseases (S1B Fig in S1 File). The node size reflects the change in degree value size, the larger the node the larger the degree value, where Stat3 has the largest degree value, therefore, it is hypothesized that among the above core targets, the sustained activation of signaling represented by the Stat3 target is closely related to cancer development. The results of Kyoto Encyclopedia of Genes and Genomes (KEGG) [68] pathway enrichment analysis showed that the most involved disease signaling pathways of the core targets were mainly cancer-related pathways and viral infection pathways, including bladder cancer, pancreatic cancer, hepatitis B and tuberculosis. The final network topology results were analyzed to conclude that the active ingredients of ginger-licorice formula are mainly used to treat cancer diseases by acting on 64 targets such as Stat3, Jun, TP53, and TNF (degree greater than twice the median is the core target). For the 64 core targets screened, this study further explored the 18 main active ingredients of the formula for cancer treatment. Compound target analysis identified six active ingredients corresponding to a number of targets greater than the median value of degree, namely quercetin, isorhamnetin, kaempferol, licorice chalcone A, and naringenin. Of these, only quercetin corresponded to the above four core targets and had the same maximum degree value. Notably, these cancer pathways are linked to both small molecule quercetin and target Stat3. Based on the results of network pharmacology, anticancer mechanism of the bupleurum-ginger-Licorice formula was preliminarily proposed: quercetin down-regulated Stat3 signal and gene expression by interfering phosphorylation.

**Fig 3:**
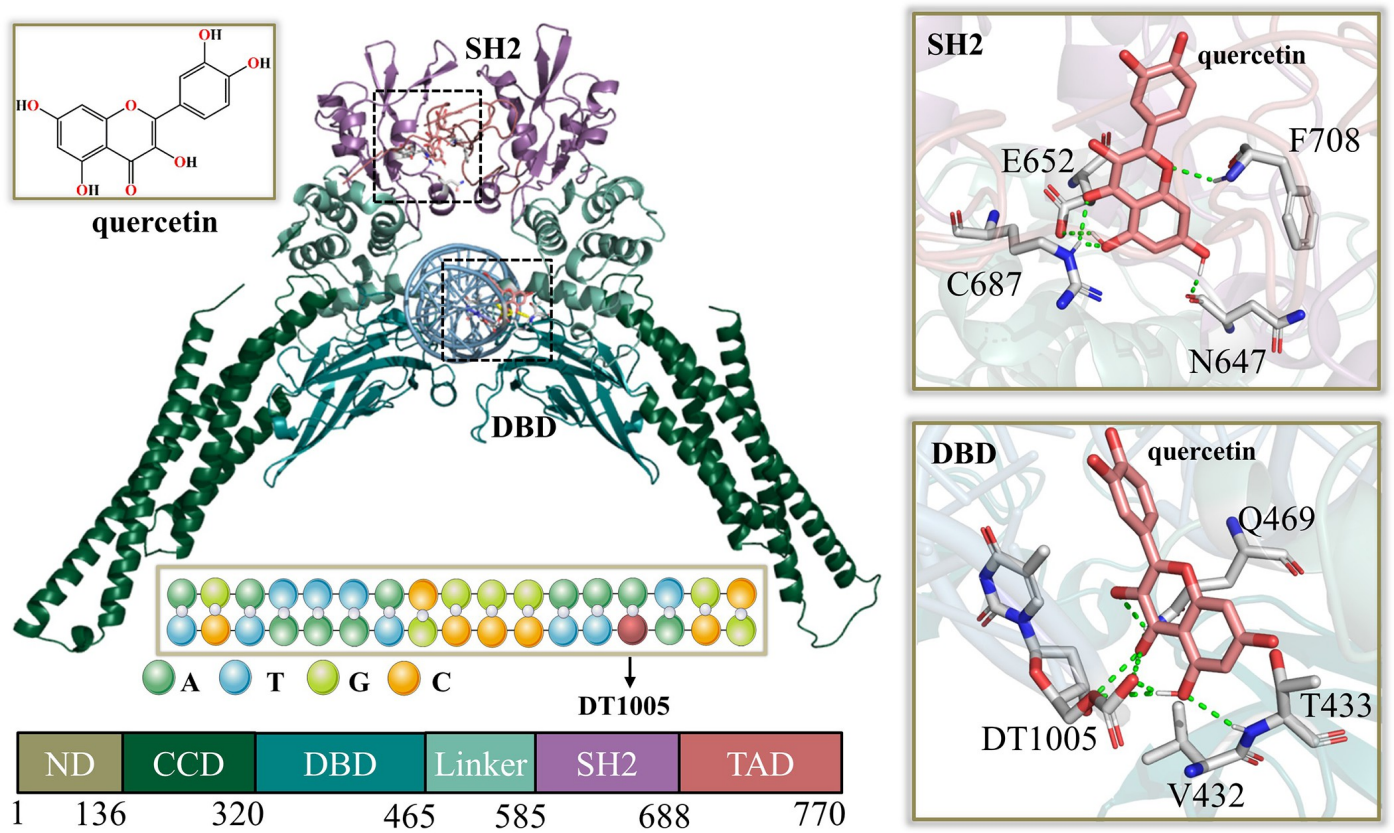
Two representative binding modes between quercetin and Stat3.

According to the similarity property principle (SPP), compounds with similar structures have similar biological activities and act on the same targets. In this work, molecular similarities between quercetin and a series of representative anticancer drugs were analyzed to determine its possible targets and anticancer mechanisms. S2 Fig in S1 File shows 24 representative anticancer inhibitors acting on four potential anticancer targets (i.e., Stat3 [69,70], Jun [71], TP53 [72] and TNF [73]) screened by network pharmacology. As seen from Fig 3, quercetin and compounds 20-24 have high shape similarity of over 0.7, indicating that quercetin and these inhibitors have similar chemical properties and action mechanism. In fact, quercetin and compounds 20-24 both have the same flavonoid parent structure, which strongly supports Stat3 as an anticancer target of this small molecule.

Through network pharmacology we obtained two small molecules related to the target Stat3, quercetin and licochalcone A. Both small molecules are of interest, but in the present work quercetin is the focus of the study.

### The acquisition of quercetin-Stat3 complex

Based on molecular docking results, Fig 3 shows two representative binding modes of quercetin to the SH2 and DBD domains of Stat3 (PDB ID: 6QHD) [72,74] respectively. As mentioned above, the SH2 region dominates the dimerization of Stat3, while the DBD region is responsible for molecular recognition with substrate DNA. The formation of Stat3 dimer and its recognition with substrate DNA occur in cytoplasm and nucleus, respectively. In order to be consistent with the actual physiological process, the structures of quercetin bound to the SH2 domain of Stat3 dimer (i.e., Stat3_Lig) and to the DBD domain of Stat3-DNA complex (i.e., Stat3_DNA_Lig) both were acquired for subsequent conformational change and molecular recognition analyses. In the SH2 domain of Stat3, the inhibitor forms H-bonds with N647, E652, F708 and C687; while for DBD domain, V432, T433, Q469 were mainly involved in H-bond formation. Quercetin also has a stable H-bond with the DT1005 (DT23) base of substrate DNA, which tentatively suggests that the association with the inhibitor may affect the transcriptional efficiency of Stat3, which will be discussed in detail in the following DNA conformational analysis.

### Convergence of simulated trajectories

Four 100 ns comparative MD simulations were performed for the systems Stat3, Stat3_Lig, Stat3_DNA, Stat3_DNA_Lig. S3 Fig in S1 File shows the convergence parameters including potential energy, root mean standard deviation (RMSD), root mean standard fluctuation (RMSF) in the simulation process. The Stat3, Stat3_Lig, Stat3_DNA, Stat3_DNA_Lig systems all achieve energy equilibrium after 5 ns, which is a prerequisite for subsequent studies of conformational change and molecular recognition. As shown in S3B Fig in S1 File, the RMSD values of Cα atoms in four systems (i.e., Stat3, Stat3_Lig, Stat3_DNA, Stat3_DNA_Lig) are 4.785±0.014, 6.009±0.017, 3.489±0.008 and 3.862±0.009 Å, respectively. Among them, the increase in RMSD value is mainly due to the elimination of crystal stacking and conformational transitions, while the decrease in RMSD value is due to the repair of inappropriate folds and the formation of a new locally stable conformation. In comparison, molecular stability of Stat3 is improved after binding to endogenous DNA. When the inhibitor quercetin binds to SH2 and DBD domains, the intrinsic topological structure of Stat3 is partially affected, and the overall molecular flexibility is slightly enhanced. By comparing the RMSF, the flexible distribution at the residue level was basically the same for the four systems (see S3C Fig in S1 File). In addition, the RMSF correlation coefficient of 0.91 between Stat3_DNA and Stat3 also proves the reliability of the simulated trajectories (see S3D Fig in S1 File).

### Conformational change of Stat3 induced by quercetin binding

As Stat3 binds to DNA and initiates the transcription process, the recognition region maintains a broad three-dimensional space that is essential for biochemical reactions. Since DNA binds to the DBD (F321-S465) of the Stat3 dimer, the time-dependent distance between the centroids of two DBDSs is measured here, which can partially characterize the size of the substrate DNA binding pocket. As shown from S4 Fig in S1 File, the distances between DBDs in the two DNA-bound Stat3 systems maintain stable at around 15 Å, indicating that all the MD simulations are stable and reliable. With the association of quercetin, the distance between DBDs becomes narrower, which partially limits the freedom of movement of the substrate DNA and reduces its transcriptional efficiency.

**Fig 4:**
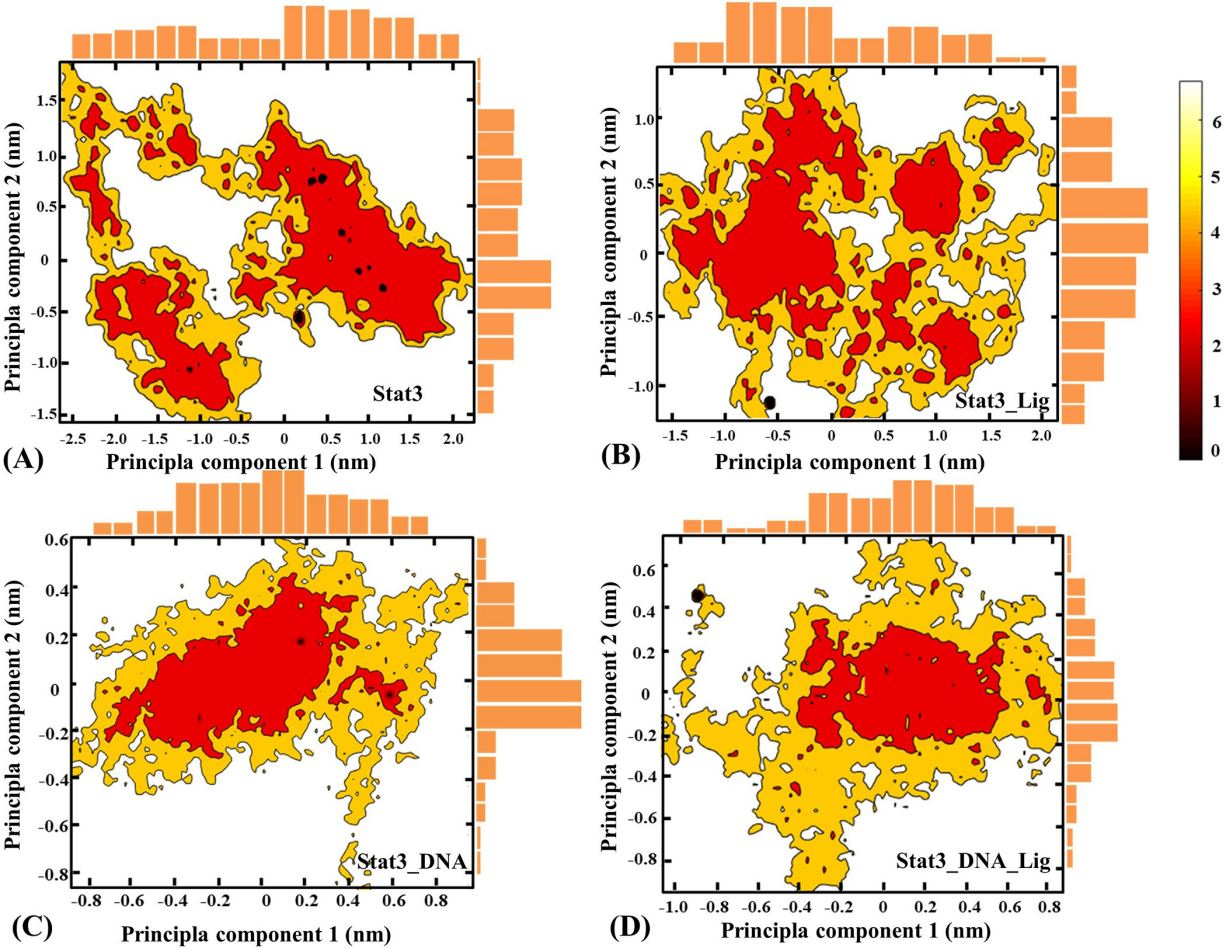
Free energy landscapes versus the first two PCs in the Stat3(A), Stat3_Lig(B), Stat3_DNA(C) and Stat3_DNA_Lig (D) systems.

In addition to RMSD, RMSF and distance function analyses for global conformational distribution, Free Energy Landscape (FEL) based on Principal Component Analysis (PCA) and probability statistics can be used to capture fluctuation amplitude and conformational changes of functional motions. Fig 4 shows the FEL in the Stat3, Stat3_Lig, Stat3_DNA and Stat3_DNA_Lig systems at 300 K, with deeper color indicating lower free energy. The following two viewpoints can be obtained: (1) Stat3 conformation becomes more compact and structural distribution area is smaller after DNA binding; while the association with quercetin has relatively little influence on the transition range of the system; (2) The conformational distribution of Stat3_DNA and Stat3_DNA_Lig is similar, which indicates that quercetin binding only affects the local conformation rather than the whole protein structure in DNA-bound systems. While in the DNA-free Stat3 system, quercetin binding results in a change from discontinuous to continuous distribution, which obviously involves the global movement of molecules.

**Fig 5:**
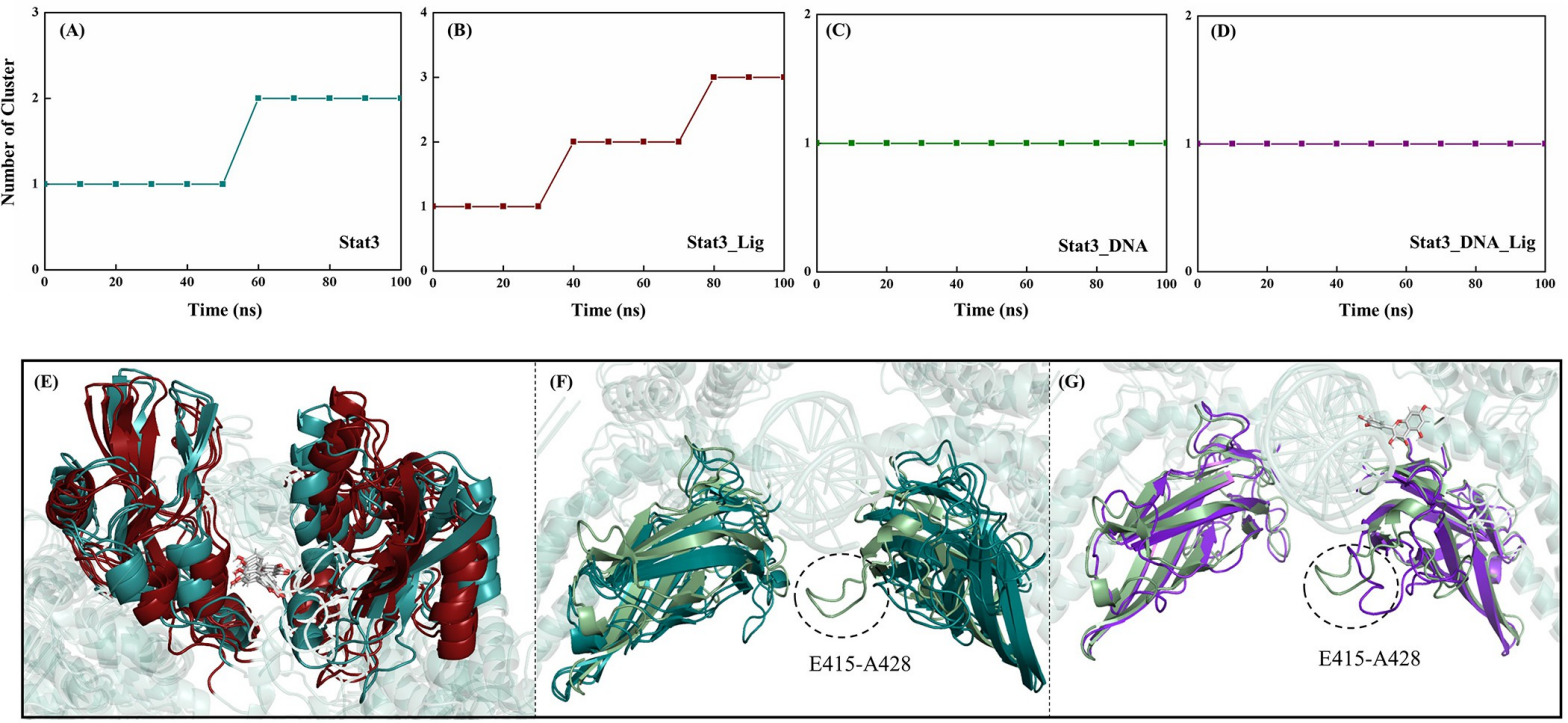
The number of clusters in the Stat3 (A), Stat3_Lig (B), Stat3_DNA (C) and Stat3_DNA_Lig (D) systems over simulation time, as well as the conformational superimposition for the SH2 (E) and DBD (F, G) domains.

The previous RMSD and RMSF analyses tentatively show that the molecular flexibility from high to low is Stat3_Lig > Stat3 > Stat3_DNA_Lig ≈ Stat3_DNA. According to the above FEL analysis, the association with quercetin affects the global conformation of DNA-free Stat3, as well as the local conformation of DNA-bound Stat3. In order to intuitively understand how the binding of quercetin affects the structure of Stat3 and Stat3_DNA, Conformational cluster analyses were performed based on MD trajectories of the four systems (i.e., Stat3, Stat3_Lig, Stat3_DNA and Stat3_DNA_Lig), and their representative conformations in each cluster were superimposed (see Fig 5). As shown from Fig 5A–5D, the cluster numbers of the Stat3, Stat3_Lig, Stat3_DNA and Stat3_DNA_Lig systems are 2/ 3/ 1/ 1, respectively, which is consistent with the number of low free energy regions of FEL. Fig 5E–5G shows the representative conformation with low free energy for SH2 and DBD domains in different clusters. After quercetin binding, the global conformation of Stat3 underwent significant changes, especially the interfacial α-helix (E592-S599 and I653-D661) became disordered, which was not conducive to dimer formation and thus down-regulated the biological function of gene transcription (see Fig 5E). Compared with Stat3, the Stat3_DNA system become more stable: the cluster number of the whole conformation changes from 2 to 1 with a local conformational change occuring in the loop E415-A428 (see Fig 5F). As shown from Fig 5G, the dihydroxyphenyl group of quercetin is vertically embedded into DNA major groove and Stat3 DBD (F321-S465) narrowing their spatial distance, which is not conducive to providing large space required for DNA transcription.

### Molecular recognition

Quercetin binds to two sites on Stat3, namely SH2 and DBD. The H-bond, binding free energy, weak interactions and conformational change were analyzed for quercetin-bound complexes (i.e., Stat3_Lig and Stat3_DNA_Lig).

#### Analyses of intramolecular H-bonds

H-bonds play an important role in maintaining the structural stability of the protein and the specific recognition of the receptor ligand. Table 1 lists the inter-monomer and DBD-DNA H-bonds formed by SH2, DBD, DNA, quercetin for the Stat3, Stat3_Lig, Stat3_DNA, Stat3_DNA_Lig systems. H-bond formation is determined by geometric criteria, i.e. donor-H-acceptor angle greater than 135° and donor-acceptor distance less than 0.35 nm. The number of H-bonds, whether total or occupying more than 30%, is Stat3_DNA_Lig > Stat3_DNA > Stat3 > Stat3_Lig in descending order. The possible reasons behind three greater-than signs are as follows: the association of quercetin with DBD makes the pocket more compact, bringing hindrance to gene transcription; the introduction of DNA results in a large increase in total atomic number; quercetin binding to SH2 region widens the distance between Stat3 monomers, not conducive to dimerization. As listed in Table 1, there are three H-bonds between monomers, with all of which distributed in SH2 of Stat3 dimer, while decreasing to one in Stat3_Lig. Quercetin can’t act as an intermediate bridge connecting two monomers, it only forms the H-bond with E638 of chain A, while it doesn’t interact with chain B. In a word, quercetin binding to SH2 reduces the H-bond between monomer interfaces, which may weaken the stability of Stat3 dimer.

**Table 1:** The inter-monomer and DBD-DNA H-bonds formed by SH2, DBD, DNA, quercetin for the Stat3, Stat3_Lig, Stat3_DNA, Stat3_DNA_Lig systems.

Systems	H-bond (%)	Acceptor	Donor
Stat3	812	Q644, Q508^’^, N647	N647^’^, Q508^’^, Q509^’^
Stat3_Lig	797	Lig, C687^’^	E638
Stat3_DNA	818	H332, Q344, R382, R417, S465	DG11, DG10, DT25, DC26, DT25
Stat3_DNA_Lig	844	H332, R382, R417, Lig, V432, S465	DG10, DT22, DT23, DT24, DT25,

H-bond (%) indicates the number of hydrogen bonds with an occupancy of 30% or more. The apostrophe in the table identifies the chain B of Stat3 dimer.

Because of the complete symmetry between chains A and B of the Stat3 dimer, the DNA transcription that occurs in the DBD region, as well as quercetin being bound between DNA and enzyme chain A, only the H-bonds formed among Stat3 chain A, DNA and quercetin are compared (see Table 1 and S5 Fig in S1 File). In Stat3_DNA, five H-bonds are formed between Stat3 and DNA, all of which are distributed in the DBD region; Quercetin was chelated by DT22 and DT23, and the accompanied DNA rotation of 36 degree (exactly one base rotation angle) resulted in the global recombination of DBD-DNA H-bonds; however, the key residues for DNA identification are still H332, R382, R417, and S465. It is consistent with the previous results of Verdura et al., that is, a series of flavonoid molecules can specifically recognize both base DT23 and residue V432 in Stat3_DNA [75]. In sum, quercetin binds to the DBD region, narrowing the DBD-DNA distance and reducing the free space required for gene transcription, which is consistent with the previous result that the inter-DBD centroid distance becomes closer.

#### Binding free energy calculation

Binding free energy can be used to effectively evaluate receptor-ligand recognition, which has become the best criterion for lead selection in drug design. Table 2 lists binding free energies and their various energy terms for the Stat3, Stat3_Lig, Stat3_DNA and Stat3_DNA_Lig systems. Quercetin can bind to two sites in Stat3——the SH2 region promoting protein dimerization, and the DBD region, which is responsible for DNA recognition. Here, the binding free energy between Lig-Sa/Sb (-4.06 kcal·mol^-1^) was higher than that between Lig-Sa/Sb/DNA (-6.74 kcal·mol^-1^), indicating that quercetin was more likely to bind to DBD region. By investigating the effect of quercetin on epithelial-mesenchymal transition (EMT) and invasion of pancreatic cancer cells using cell experiments, Yu et al. found that it significantly reduced the increase of Stat3 phosphorylation caused by IL-6 with IC50 of 80 M (Δ*G*_exp_= -5.66 kcal·mol^-1^) [76]. As listed from Table 2, the predicted free energy values of Sa-Sb and Sb-Sa/Lig are on both sides of Δ*G*_exp_, which fully proves the credibility of the calculated data (***Δ****G*_cal_). Given that binding free energy between Sa-Sb (-7.7 kcal·mol^-1^) is less than that between Sb-Sa/Lig (-5.76 kcal·mol^-1^), quercetin binds to Stat3 interface and partially inhibits its dimerization. In addition, binding free energy between DNA-Sa/Sb (-8.98 kcal·mol^-1^) was lower than that between DNA-Sa/Sb/Lig (-7.87 kcal·mol^-1^), indicating that quercetin binds to the DBD region and weakens its original stable interactions with DNA, being not conductive to subsequent STAT3-dominated DNA transcription.

**Table 2:** The composition and contribution of each energy item to binding free energy (kcal·mol^-1^).

Items	Sa-Sb	Lig-Sa/Sb	Sb-Sa/Lig	DNA-Sa/Sb	DNA-Sa/Sb/Lig	Lig-Sa/Sb/DNA
*Δ*ELE_IN_	-261.58±3.62	-32.86±3.31	-107.46±3.01	-3700.31±5.15	-3665.93±5.04	-68.71±5.44
*Δ*VDW_IN_	-124.30±0.25	-113.68±0.48	-124.44±0.44	-133.36±1.21	-110.02±0.98	-2.34±2.35
*Δ*ELE_PB_	323.71±0.33	87.64±2.51	175.62±2.87	3734.15±3.68	3675.59±10.84	51.08±2.08
*Δ*VDW_PB_	-15.16±0.05	-13.89±0.04	-15.12±0.03	-18.37±2.78	-16.89±2.71	-3.30±3.18
ΔH	-77.33±0.33	-72.80±1.21	-75.41±2.70	-117.90±3.55	-117.24±11.83	-23.28± 1.14
TΔS	-69.61±10.50	-68.74±10.64	-69.65±6.30	-108.92±6.30	-109.37±5.04	-16.54±5.21
*ΔG*_cal_	-7.7	-4.06	-5.76	-8.98	-7.87	-6.74

**ΔELE_IN_** stands for the sum of intramolecular non-bonded electrostatic energy and 1,4-electrostatic energy; **ΔVDW_IN_** is used to indicate for the sum of intramolecular non-bonded van der Waals energy and 1,4-van der Waals energy; **ΔELE_PB_** refers to solvation electrostatic energy calculated with PB algorithm; **ΔVDW_PB_** is solvant van der Waals energy calculated with PB algorithm; **Δ*H*** and ***T*Δ*S*** respectively represent enthalpy and the product of absolute temperature with entropy; Δ***G***_cal_ is the calculated binding free energy, obtained from **Δ*H*** - ***T*Δ*S***. **SA-SB** represents the binding free energy between monomers of Stat3 dimer; In the Stat3_Lig system, **Lig-Sa/Sb** and **Sb-Sa/Lig** respectively indicate binding free energies between quercetin and Stat3 dimer, as well as between chain-B and chain-A/quercetin; **DNA-Sa/Sb** represents the binding free energy between DNA and Stat3 in the Stat3_DNA system; In the Stat3_DNA_Lig system, **DNA-Sa/Sb/Lig** and **Lig-SA/Sb/DNA** respectively show binding free energies between DNA and Stat3/quercetin, as well as between quercetin and Stat3/DNA.

We calculated the binding free energies of the Stat3_Lig and Stat3_DNA_Lig systems using the SIE method, and the corresponding values of the binding free energies and each energy are shown in Table 3.

**Table 3:** Binding free energies and energy components calculated by SIE method.

	Δ*E*_*VDW*_	Δ*E*_*C*_	$\Delta G_{bind}^{R}$	*γ*Δ*MSA*	Δ*G*_*bind*_	Δ*G*^*exp*^
Stat3_Lig	-20.92±1.53	-16.69±1.17	14.70±0.72	-3.92±0.6	-5.46±0.11	-5.66
Stat3_DNA_Lig	-22.19±1.77	-23.17±1.63	19.44±0.70	-5.12±0.05	-6.14±0.11	—

Δ*E*_*VDW*_ is the molecular van der Waals force; Δ*E*_*C*_ is the molecular Coulomb force interaction; $\Delta G_{bind}^{R}$ is the change in the reaction field energy; in *γ*Δ*MSA*, Δ*MSA* is the change in the molecular surface area, *γ* is the molecular surface area coefficient (0.0129 kcal/mol·A^2^); Δ*G*_*bind*_ is the binding free energy.

Among all the energy terms, the intervention of DNA makes the system more stable, as can be seen from the data in the table, Δ*E*_*VDW*_, Δ*E*_*C*_, $\Delta G_{bind}^{R}$ and *γ*Δ*MSA* favor the binding of the inhibitor and Stat3, suggesting that quercetin binds to the DBD region, weakening its pre-existing stabilizing interactions with the DNA and disrupting the subsequent Stat3-dominated DNA transcription. Δ*E*_*VDW*_ and *γ*Δ*MSA* are nonpolar interactions and Δ*E*_*C*_ and $\Delta G_{bind}^{R}$ are polar interactions. A synthesis of these two interacting forces can be found: Enhanced non-polar interactions due to the large contact area between DNA and Stat3, At the same time quercetin is sandwiched between DNA and protein, tightly coupling these two interactions and thus favoring protein-inhibitor interactions; Then again, the increase in polar interactions also suggests that transcription of DNA is difficult to occur when STAT3 and quercetin bind. Comparison of the different energy values shows In the Stat3_Lig system, the predicted values are more in line with the experimental values, and the binding of quercetin disrupts the formation of dimers; In the Stat3_DNA_Lig system, the increase in each value indicates that the protein as a whole is much more stable, and that it is difficult to transcribe the DNA after the intervention of quercetin.

#### Affecting DNA weak interactions and conformation

**Fig 6:**
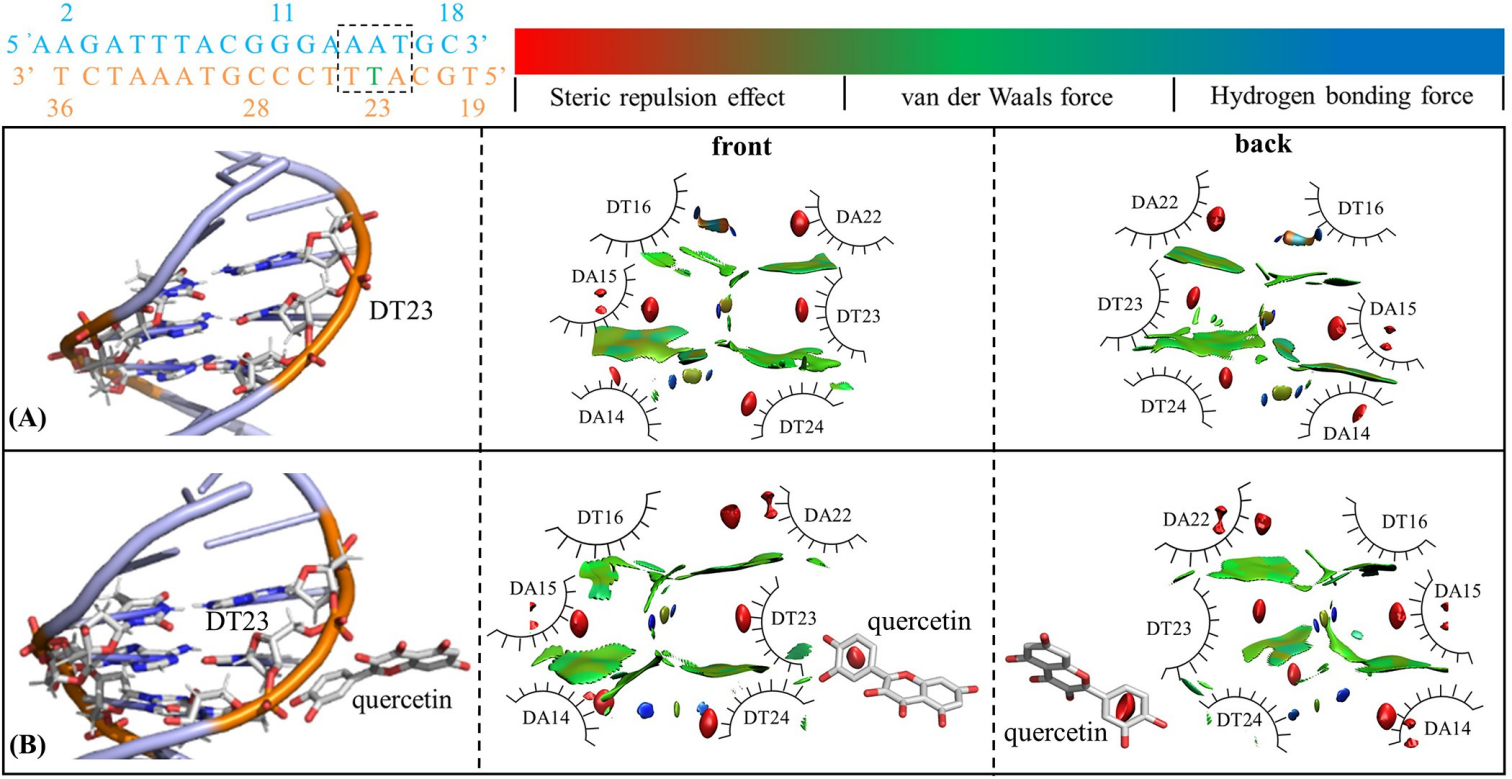
The weak interaction analysis of DNA at quercetin binding sites in the Stat3_DNA (A) and Stat3_DNA_Lig (B) systems.

Although quercetin binding does not affect the overall motion pattern of DNA, what effect does it have on the local conformation? Weak interaction analysis based on electron density function is a common method to explore the mechanism of DNA local conformational change. From the MD trajectories of Stat3_DNA and Stat3_DNA_Lig, the effects of quercetin binding on the mean weak interaction of DNA were comparatively analyzed. With the lowest energy conformation used as the display frame, DA15P and DT23P atoms both were selected as the center for visual weak interaction analysis. Fig 6 shows the weak interaction analysis of DNA at quercetin binding sites in the Stat3_DNA and Stat3_DNA_Lig systems from three perspectives. Due to the large number of aromatic rings in the bases, the DNA portion of Stat3_DNA system showed typical van der Waals interactions represented by green patches, especially π-π stacking effect. As shown from Fig 6, with the binding of quercetin (i.e., the Stat3_DNA_Lig system), the aromatic ring of DT23 changes from its conventional position to almost perpendicular to the plane of quercetin. The π-π stacking effect between DT23 and adjacent bases was significantly reduced, nevertheless partially compensated by slightly enhanced H-bond interactions. In addition, quercetin showed moderate van der Waals interactions and strong steric hindrance effect with both DT23/ DT24. In conclusion, the binding of quercetin weakens the regular van der Waals interactions between DNA base pairs and also introduces stronger steric effect, which may attenuate the transcription efficiency of DNA.

DNA conformational parameters were obtained using the Curves software package for the Stat3_DNA and Stat3_DNA_Lig systems. S6 Fig in S1 File shows variation of four representative parameters (i.e., bend, twist, major/minor groove widths) over simulation time. The parameters of bend, twist, major/minor groove widths in the two systems did not change significantly, remaining at around 1.5, 34.5, 11.5/5.5 Å. In detail, quercetin binding reduced Bend but increased Twist values, with little overall effect on DNA flexibility, which was consistent with previous RMSD and RMSF analyses. It is worth mentioning that the time-dependent variation trends of major/minor groove widths are complementary, which fully proves the rationality of Curve analysis.

Major/minor grooves of DNA often serve as binding sites for proteins, and their widths can directly characterize protein-DNA molecular recognition. S7A Fig in S1 File shows variations of average major/minor groove width values at the base level for the Stat3_DNA and Stat3_DNA_Lig systems. At the site of DA13-DT16/DA22-DT25 of DNA, the larger width of the major groove was favorable for the association of quercetin, while the corresponding width of the minor groove was smaller. With the binding of quercetin, the major groove width of DA13-DT16/DA22-DT25 continued to increase, accompanied by the synergistic reduction of the major groove width of DT5-DT7 /DA31-DA33 and almost no change in the width of whole minor groove. It also indicates that the Stat3 complex systems with DNA (i.e., Stat3_DNA and Stat3_DNA_Lig) are generally stable, which is consistent with previous results of RMSD, FEL and Cluster. In general, major and minor groove widths should have a high negative correlation in standard B-DNA. As shown in S7A Fig in S1 File, the correlation coefficients of major/minor groove widths in Stat3_DNA and Stat3_DNA_Lig were -0.83 and -0.87, respectively. The high negative correlation coefficients also proved that the binding of quercetin had little influence on the overall conformation of DNA. The previous weak interaction analysis has suggested that the formation of quercetin-DT23 H-bond partially reduces the original Van der Waals interactions between base pairs, leading to slight local rotation and bending of DNA. Compared to Stat3_DNA, quercetin binding (i.e., Stat3_DNA_Lig) resulted in only very slight changes in local flexibility and motion of DNA (see S7B Fig in S1 File).

### Possible inhibitory mechanism

**Fig 7:**
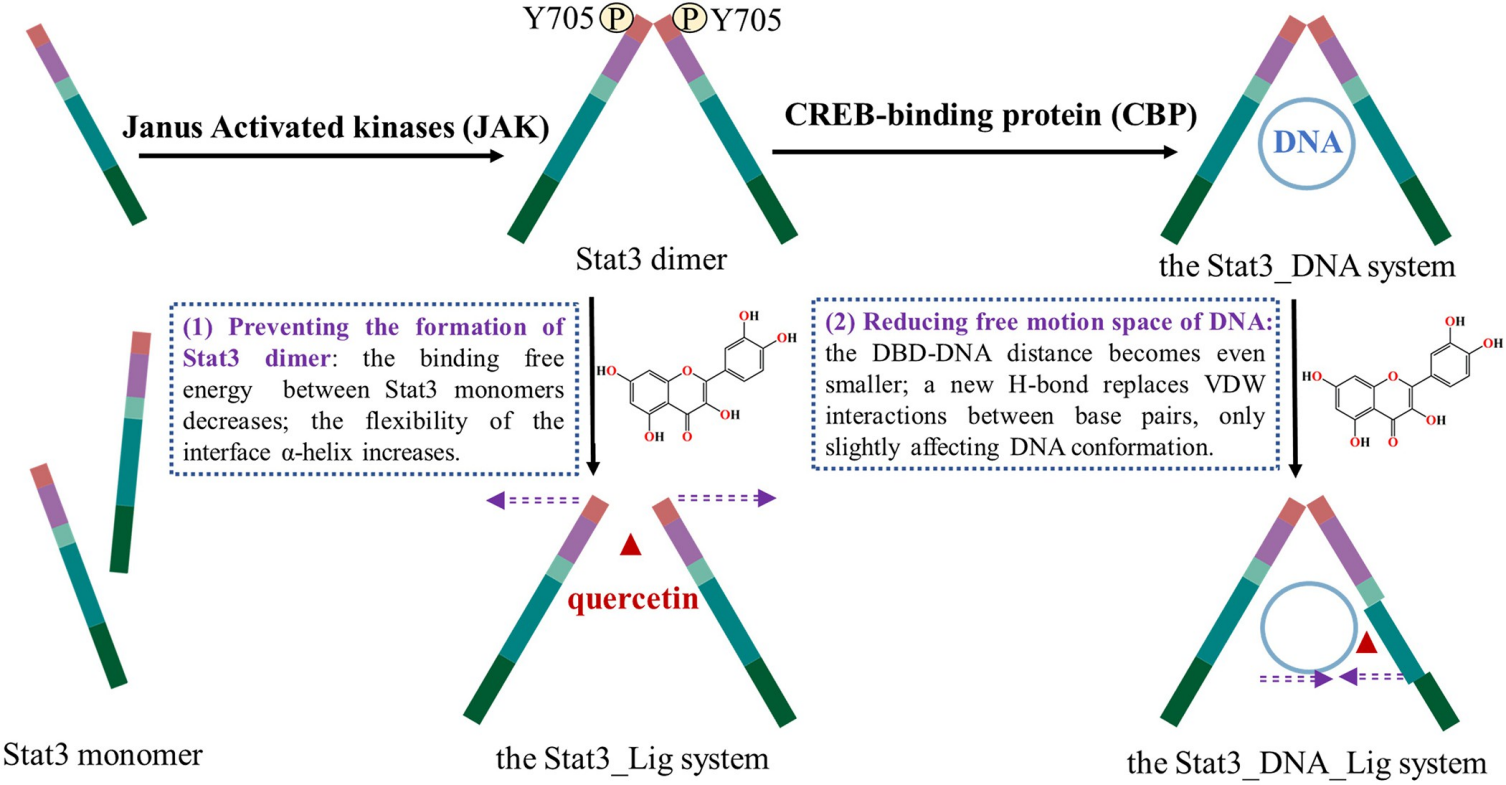
The possible inhibitory mechanism of quercetin against Stat3.

As an effective adjuvant therapy for cancer, the core functional components and targets of TCM Bupleurum-Ginger-Licorice formula have been proved to be quercetin and Stat3. Stat3 belongs to an important intracellular signaling pathway, and its continuous activation is closely related to the occurrence and development of cancer, the present work proposes a possible inhibitory mechanism of quercetin against Stat3 (see Fig 7). In response to cytokines and growth factors, Y705 in Stat3 is phosphorylated by receptor-associated Janus kinases, and the dimer is subsequently formed and translocated to the nucleus. Subsequently, K685 of Stat3 was acetylated in the nucleus by CREB binding protein (CBP) to participate in gene transcription. Small molecule inhibitors of Stat3 generally include the blockings of phosphorylation, acetylation, dimerization, nuclear translocation, and DNA association. As for quercetin, it selectively binds to SH2 and DBD regions of Stat3, showing a dual inhibitory mechanism. When quercetin binds to the SH2 region, it improves the overall molecular motility, especially that of the interface α-helix, breaks the interface H-bonds and reduces the binding free energy, ultimately inhibiting the formation of the Stat3 dimer, which is the minimum structural unit for exerting biological function. When quercetin binds to the DBD region, it forms some new H-bonds with DNA and partially replaces the original Van der Waals (VDW) interaction between base pairs, only slightly affecting the DNA structure. Moreover, the distance between DNA and DBD becomes smaller and the free motion space of DNA is reduced, which is not conducive to subsequent gene transcription.

## Discussion

There are definitely two questions in cancer biology. First, the causes of cancer are based on genetic mutations and multiple carcinogenic progressions; the second is how to treat cancer [77]? Therefore, in this paper cancer is targeted through herbal agents. In conclusion, the herbal formula of bupleurum, ginger and licorice has good ability to treat cancer, which has multi-component, multi-target and multi-pathway characteristics. Based on the network pharmacological analysis, the active ingredients quercetin, licorice chalcone and a series of possible anticancer targets of the three herbal medicines (bupleurum, ginger and licorice) were identified. However, combined with target degree values and molecular similarity analysis, the target of action of quercetin was confirmed to be Stat3.

Quercetin as a flavonoid has been shown to have anticancer activity in previous studies, is commonly found in fruits and vegetables, and is one of the most abundant dietary flavonols that promotes loss of cell viability, apoptosis, and autophagy. In the present work, the inhibitory effect of quercetin on Stat3 was systematically investigated in terms of molecular docking and molecular mimicry [78]. Molecular docking of the functional protein Stat3 obtained from network pharmacology and the key small molecule quercetin was performed to obtain ligand-containing crystal structures (dimeric structure bound to the SH2 region and DNA structure bound to the DBD region) to facilitate further molecular dynamics simulations. First, a convergence analysis was performed in which the binding of the inhibitor repaired inappropriate folds and formed a new locally stable conformation. The overall RMSD fluctuations stabilize upon DNA binding. We focused on the analysis of the distance between the two DBD regions and could see that the distance between the DBDs became larger due to the intervention of small molecules. Furthermore, the free energy surface analysis and FEL also confirmed that the entry of small molecules made the α-helix of SH2 region become disordered, which made it difficult to form the binary structure, and the entry of inhibitor for DBD region also made the β-sheet change greatly, which made the DNA unable to carry out the next transcription, further proving the inhibitory effect of quercetin.

From the hydrogen bonding of molecular recognition, the binding of quercetin to DBD makes the space more compact and brings obstruction to gene transcription; the introduction of DNA leads to a large increase in the total number of atoms; the binding of quercetin to SH2 region expands the distance between Stat3 monomers, which is not conducive to dimerization. The change in binding free energy and the inhibition of small molecules when bound to the SH2 and DBD regions were also confirmed in six systems.

Stat3 protein is a major regulator of key markers and activators of most cancers, including cell proliferation and response to DNA damage [79]. However, the transcriptional role of DNA in the DBD region may be overlooked in many studies, so the first reports of promising compounds that show the mechanism of cancer response of quinazoline ligands against DNA damage and also inhibit Stat3-induced proliferation of cancer cells demonstrate a new approach to cancer therapy [80]. Therefore, in our work we focus on the structural changes of DNA when small molecules intervene in the DBD region. We used curve to focus on the analysis of twist, bend, major groove, minor groove data and weak interactions regarding DNA, and it can be seen that the DNA undergoes some changes after the intervention of small molecules. In the weak interaction and curve analysis the bases of small molecules and DNA are polarized and pull the distance between base pairs of DNA. It can be seen that quercetin affects the conformation of DNA when it interacts with small molecules.

Early Stat3 inhibitors were mainly molecular probes, including peptides, peptidomimetics, and oligonucleotides, but their drawbacks, such as poor stability, low affinity, poor cell permeability, and low bioavailability, can limit the use of the drugs in clinical applications. Based on the functional region partitioning of Stat3, its direct inhibitors are mainly categorized into SH2, DBD structural domain inhibitors and broad-spectrum indirect inhibitors (e.g., JAK inhibitors). Firstly, in the SH2 region, direct inhibitors were developed to block protein surface interactions well, but blocking the active STAT3 dimer alone may not be sufficient to completely eliminate its signaling; When directly targeting the DBD region and disrupting its DNA-binding activity, it is possible to block the transcriptional activation function of Stat3 regardless of its activation or dimerization status, however, few small molecules have been reported as direct inhibitors of DBD to date. This is largely due to the previously perceived non-druggable nature and potentially limited selectivity of DBDs; Finally for JAK inhibitors, this usually leads to undesirable off-target effects. Therefore, in this study, a natural product inhibitor, quercetin, is proposed, and based on the calculated data, it is concluded that this flavonoid compound may be a direct inhibitor of Stat3.

## Conclusion

In this work, both network pharmacology and molecular similarity were used to determine the target Stat3 and core active ingredient quercetin for TCM bupleurum-ginger-licorice formula. The complex models of Stat3, DNA and quercetin were obtained with molecular docking experiment to explore their recognition characteristics. Subsequently, comparative MD simulations were performed for the Stat3, Stat3_Lig, Stat3_DNA, Stat3_DNA_Lig systems to provide the possible inhibitory mechanism of quercetin targeting Stat3. Molecular docking results show that quercetin specifically binds to SH2 and DBD domains of Stat3. The stable potential energy and RMSD values, as well as the high correlation of RMSF, all indicate the convergence and reliability of MD simulation trajectories of the Stat3, Stat3_Lig, Stat3_DNA and Stat3_DNA_Lig systems. According to FEL and cluster analysis, association with quercetin apparently affects the global conformation of DNA-free Stat3, increasing protein flexibility, while slightly affecting the local conformation of DNA-bound Stat3. Quercetin binds to DNA major groove and forms stable H-bonds, partially replacing the previous VDW interactions between base pairs. In addition, the major/minor groove widths still maintain a good negative correlation, demonstrating that there is only a small conformational change in the DNA. The association of quercetin reduces binding free energy between two Stat3 monomer, and the predicted results are in good agreement with experimental data. This work not only provides a possible dual inhibition mechanism of quercetin targeting Stat3: inhibition of protein dimerization and reduction of DNA free motion space both weakening transcription efficiency, but also provides useful structural information for anticancer drug design based on the structures of Stat3 and quercetin.

## Supporting information

S1 File Supplementary material contains supporting table and supporting figures. (DOCX)
