# Supplementary material for: Feasibility of the inhibitor development for cancer: A systematic approach for drug design
Source: PLoS One. 2024 Aug 22;19(8):e0306632. doi: 10.1371/journal.pone.0306632 (PMC11341021; doi:10.1371/journal.pone.0306632)
Supplement: S1 File — (DOCX) [file pone.0306632.s001.docx]

**Supplemental Material**

**Feasibility of the inhibitor development for cancer：A systematic approach for drug design**

Yu Jiang ^†^ ^a^, Ling Liu ^†^ ^a, b^, Yichao Geng^b^, Qingsong Li^b^, Daxian Luo^b^, Li Liang^a^, Wei Liu^a^, Weiwei Ouyang^b^, Jianping Hu^a*^

^a^ Key Laboratory of Medicinal and Edible Plants Resources Development of Sichuan Education Department, School of Pharmacy, Chengdu University, Chengdu, China

^b^ Department of Thoracic Oncology, Affiliated Cancer Hospital, Guizhou Medical University, Guiyang, China

^†^ These authors contributed equally to this work.

^*^ Corresponding author. E-mail address: hjpcdu@163.com.

Table S1. Codes for compounds in network pharmacology

| **Mol value** | **Code** | **Compound** | **Mol value** | **Code** | **Compound** |
| --- | --- | --- | --- | --- | --- |
| MOL000098 | A | quercetin | MOL000497 | B | licochalcone A |
| MOL000422 | C | kaempferol | MOL001645 | D | linoleyl acetate |
| MOL000449 | E | stigmasterol | MOL000354 | F | isorhamnetin |
| MOL004598 | G | 3,5,6,7-tetramethoxy-2-(3,4,5-trimethoxyphenyl) chromone | MOL004609 | H | areapillin |
| MOL013187 | I | cubebin | MOL004653 | J | (+)-Anomalin |
| MOL000490 | K | petunidin | MOL000358 | L | beta-sitosterol |
| MOL006129 | M | 6-methylgingediacetate2 | MOL001484 | N | inermine |
| MOL004328 | O | naringenin | MOL000392 | P | formononetin |
| MOL002311 | Q | glycyrol | MOL001792 | R | DFV |


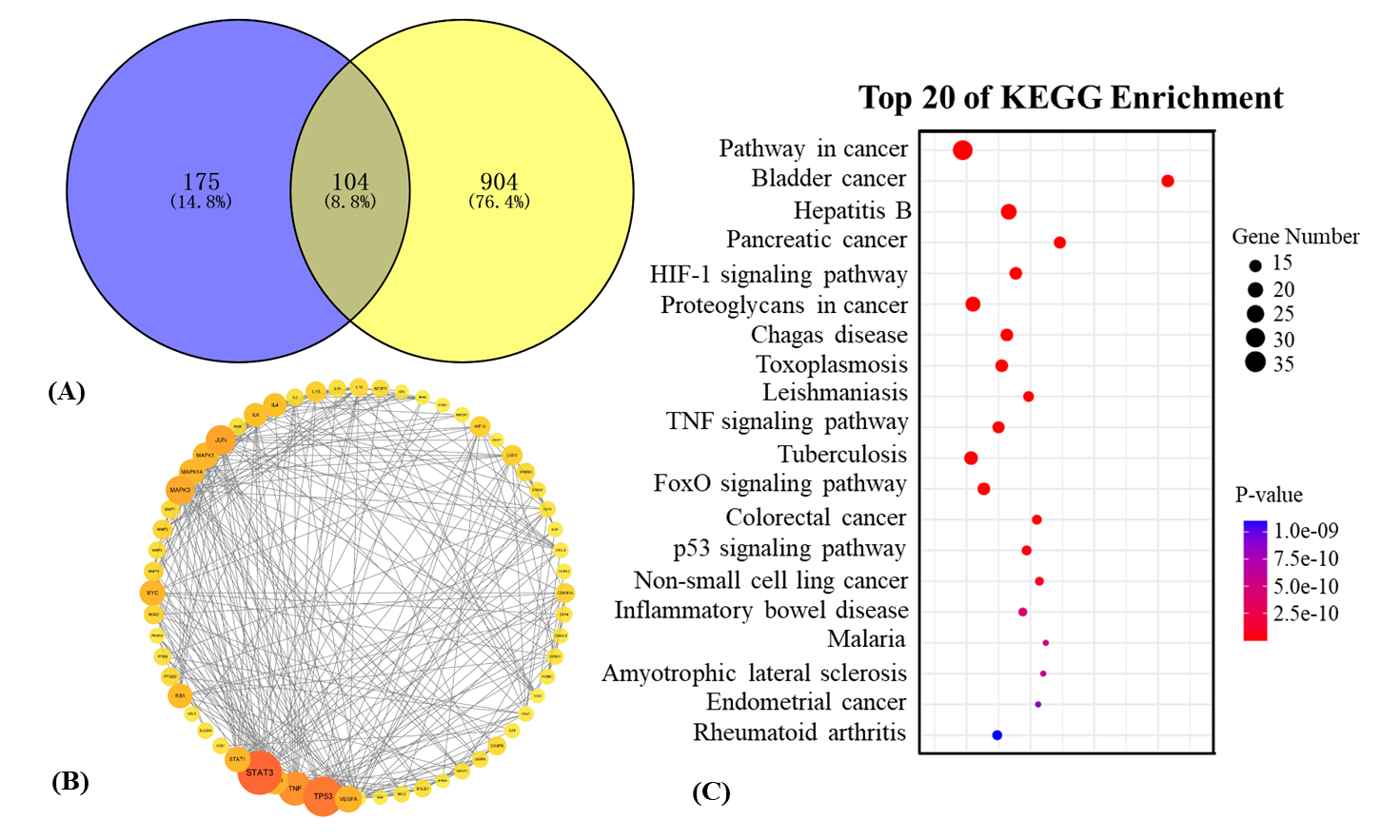


Fig S1. Genes at the intersection of disease and active ingredient (A), PPI network map (B), top 20 pathway map (C)


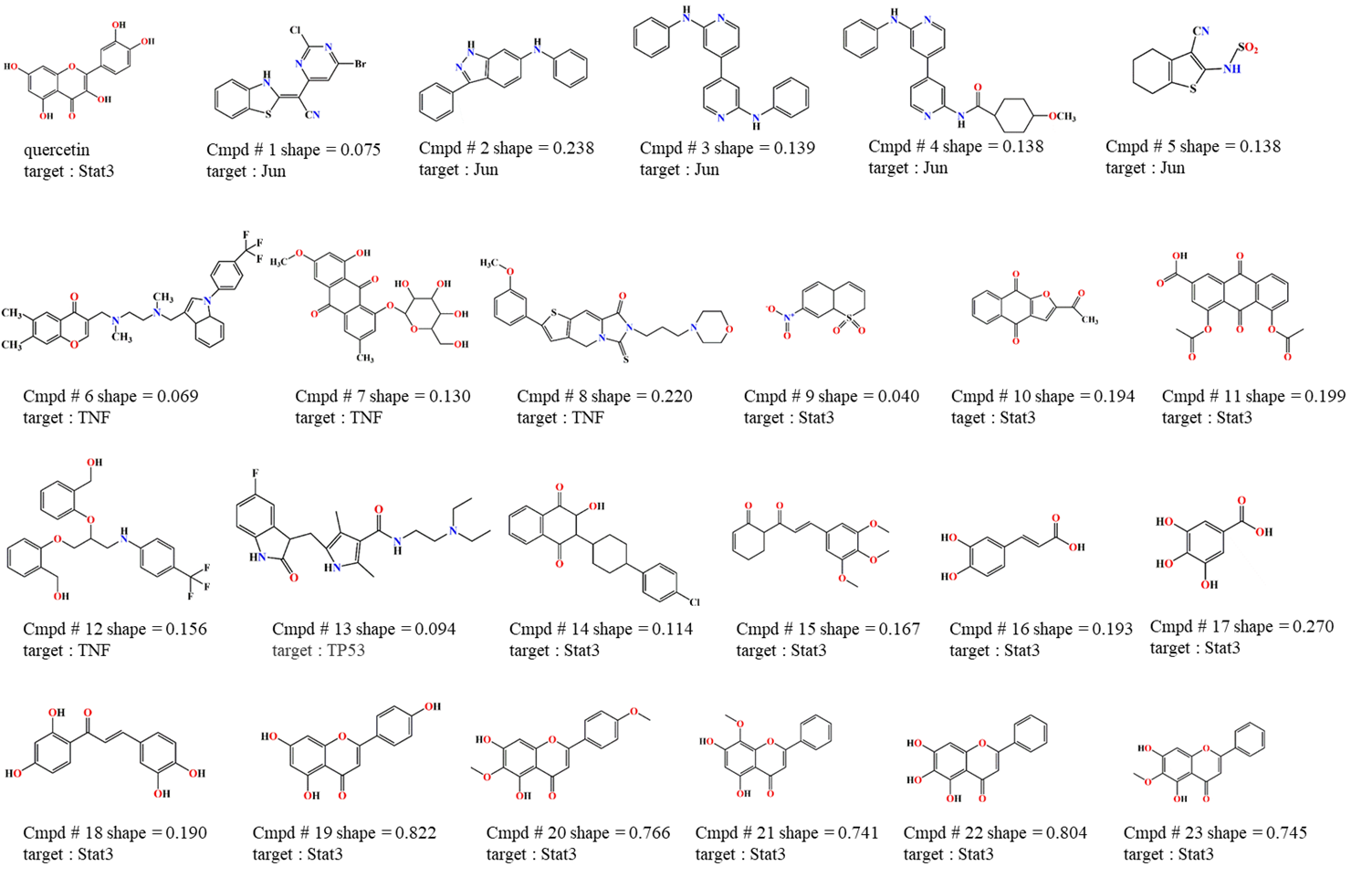


Fig S2. Molecular similarity of quercetin to 23 representative anticancer inhibitors targeting Stat3, Jun, TNF and TP53.


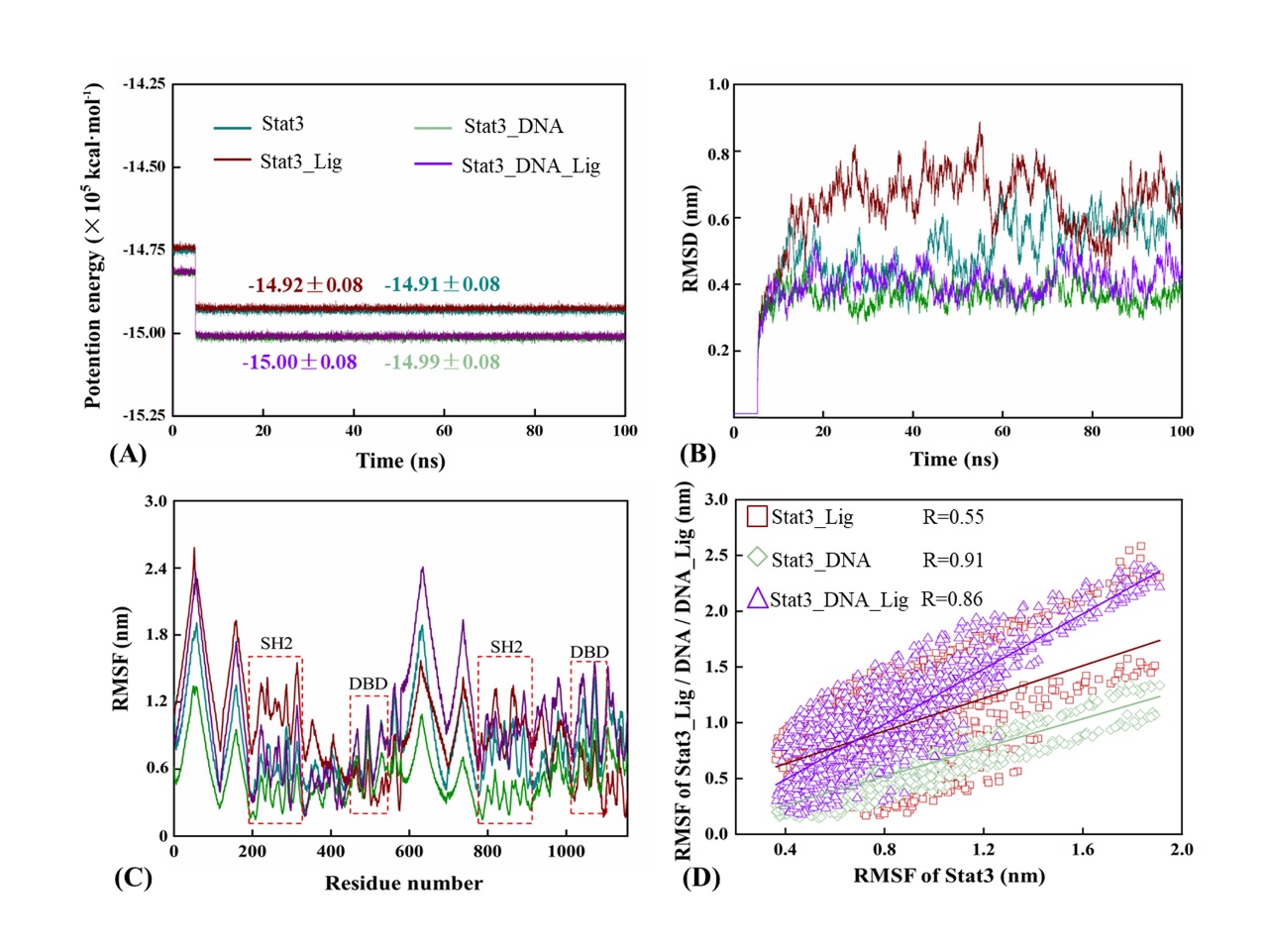


Fig S3. Comparative MD analyses of the Stat3, Stat3_DNA, Stat3_Lig and Stat3_DNA_Lig systems. Potential energy (A) and RMSD (B) over simulation time. (C) RMSF of all the Cα atoms. (D) Correlation between calculated RMSF values of Stat3_DNA and Stat3.


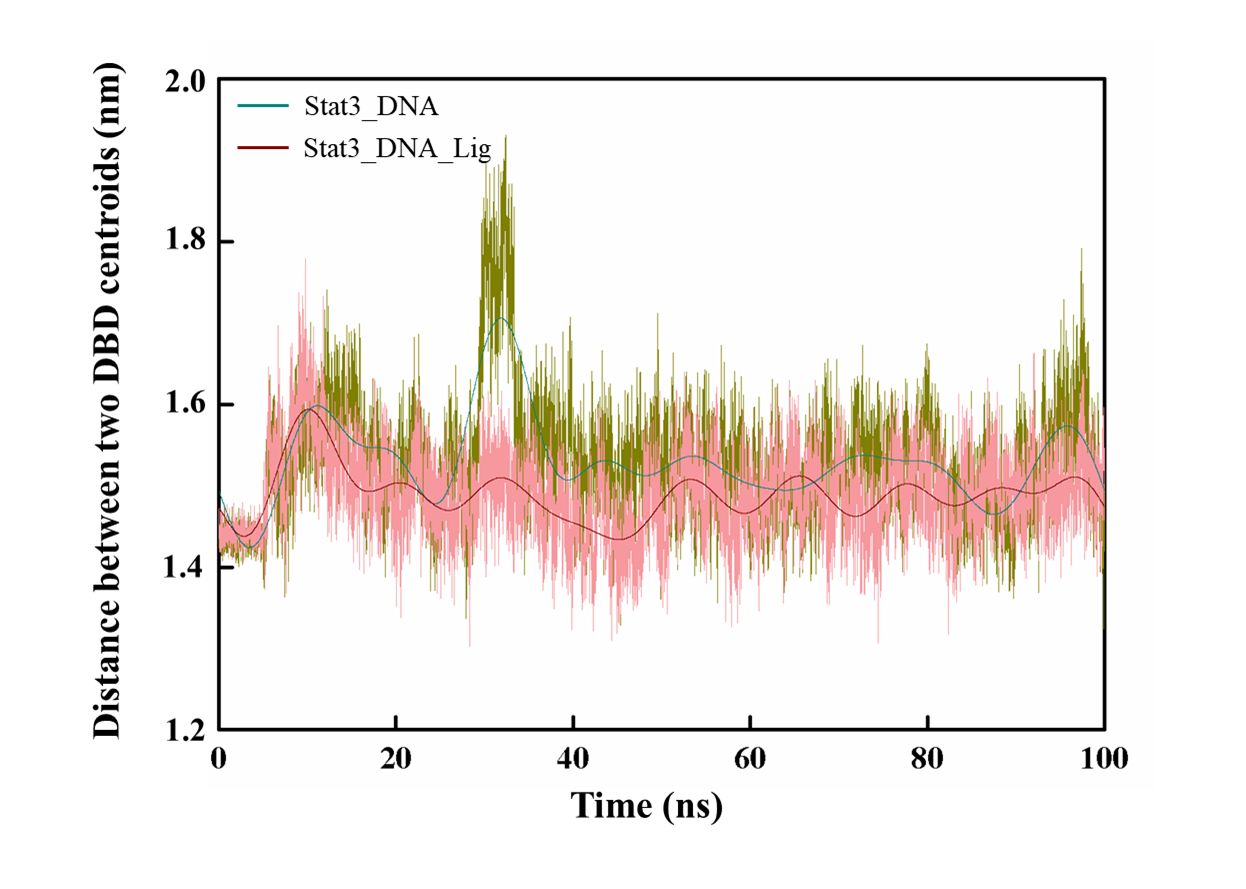


Fig S4. The distance between two DBD centroids in the Stat3_DNA and Stat3_DNA_Lig systems over simulation time.


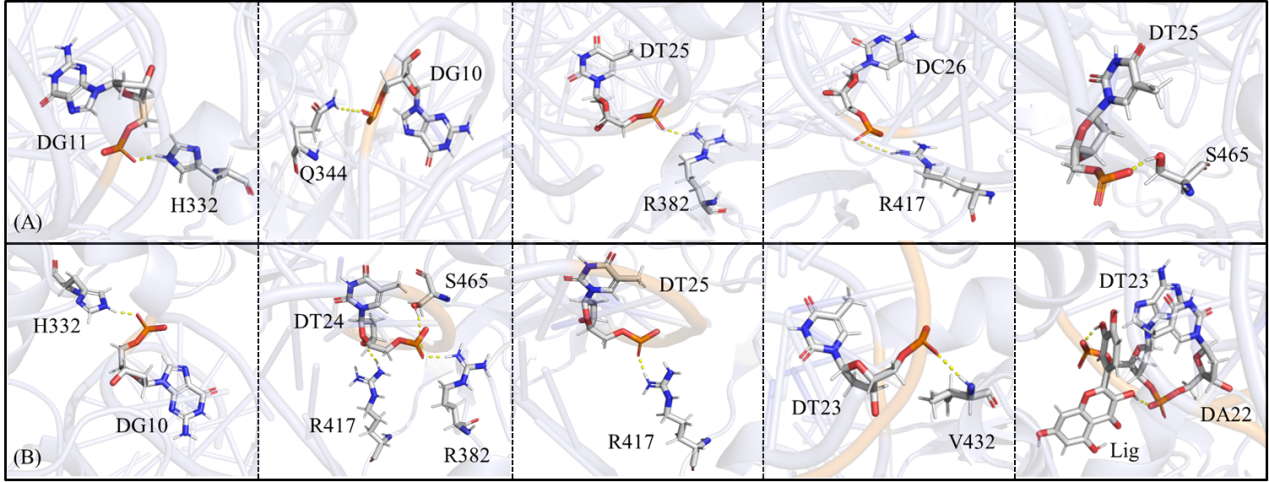


Fig S5. The DBD-DNA H-bonds in the Stat3_DNA (A) and Stat3_DNA_Lig (B) systems.


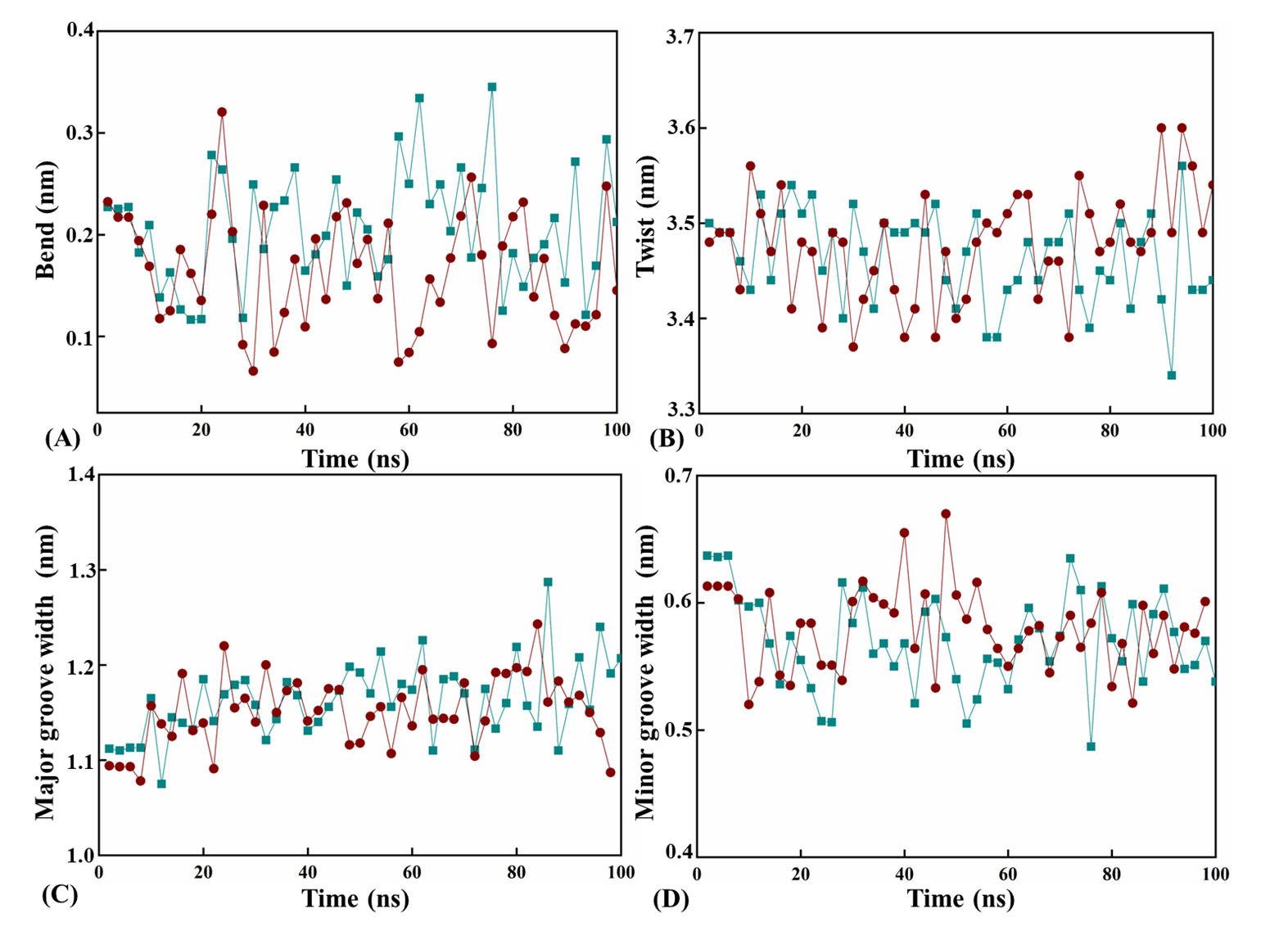


Fig S6. Variation of four DNA parameters over simulation time. (A) Bend; (B) Twist; (C) Major groove width; (D) Minor groove width.


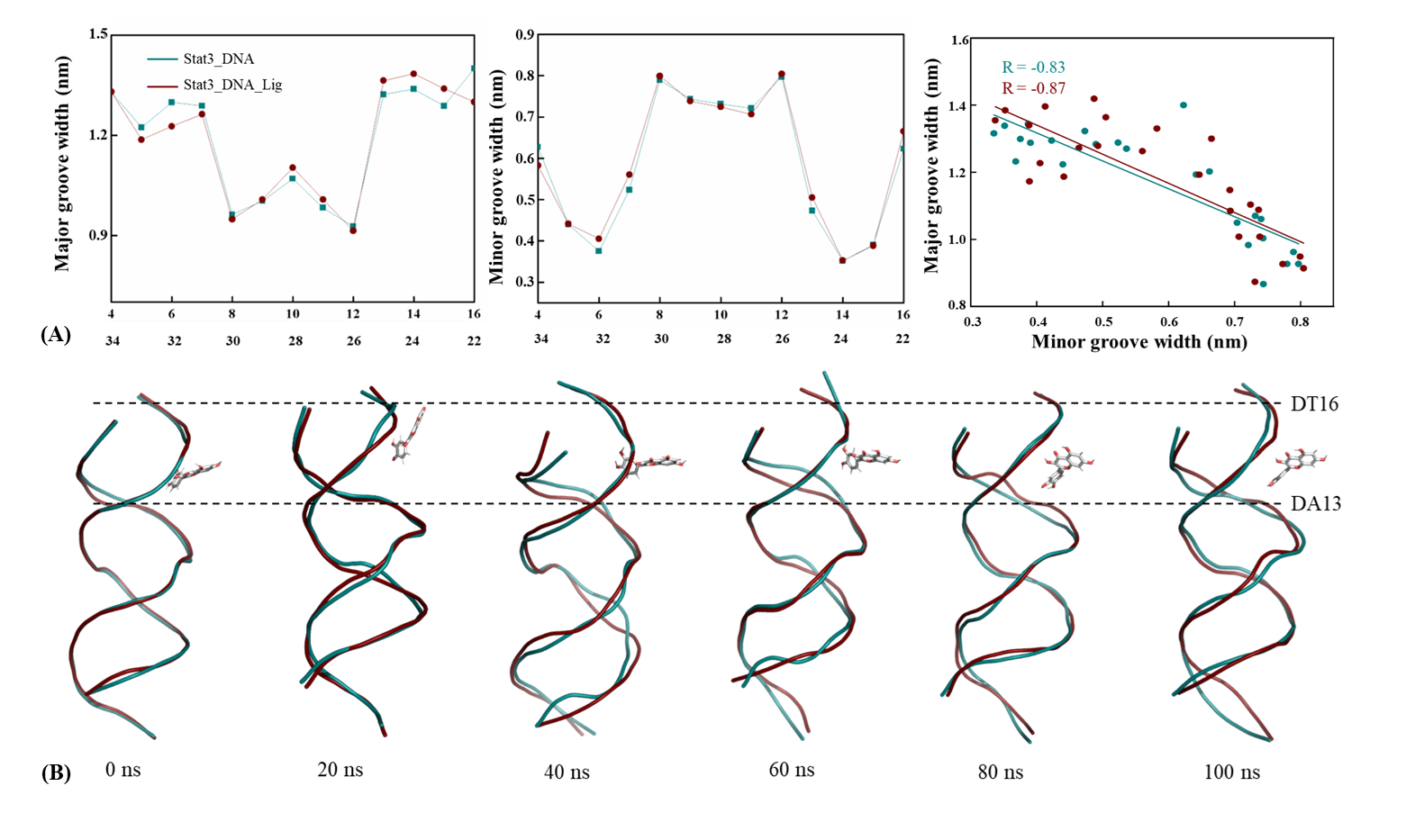


Fig S7. Effects of quercetin binding on DNA conformation in the Stat3_DNA system. (A) Average major/minor groove width values at the base level; (B) a representative conformation was selected every 20 ns based on MD trajectories of Stat3_DNA and Stat3_DNA_Lig.
